# Modified Tetanus

**Published:** 1918-01

**Authors:** 


**Modified Tetanus.** By H. BURROWS, Capt. R. A. M. C. (T. F.). Abs. from the *Lancet*, Jan. 27, 1917.

B. considers the modified form of tetanus (local tetanus), which is now the most common type resulting from war wounds, to be the natural outcome of the prophylactic injection of antitoxin.

This localized tetanus may be splanchnic, cephalic, or seated in the limbs. B. explains its occurrence on the basis of laboratory experiments with animals which have shown that the tetanus toxin gains access to the nervous system either by the general blood stream or by the motor nerves. The injection of antitoxin cuts off the approach through the general blood stream and therefore the toxin finds access only along the motor nerves. Hence it can cause only local spasticity.

Various cases illustrating the forms described are given in detail. *Splanchnic* tetanus occurred in a patient with a bad intestinal wound. 500 units of serum had been administered preventively and laparotomy with suture of the bowel performed; but 11 days later tetanus appeared : first the jaw became stiff and there was a definite trismus. Pharyngeal spasm was a pronounced feature. There was no opisthotonos. Death occurred in 8 1/2 hours after the first symptom.

A case of *cephalic* tetanus resulted from a wound penetrating to the temporo-sphenoidal lobe of the brain through the left temporal bone. On the 5th day after the injury the skull was opened and the projectile removed. 6 days later tetanus developed in spite of the injection of 750 units given shortly after the receipt of the wound. Clonic spasm of the left facial muscle and trismus were the first symptoms. Incessant masticatory movements of the jaw, lips, and tongue set in, followed by paralysis of the left facial muscles and spasms of both sides of the face. The left sterno-mastoid also became spasmodic. After 16 days the spasms ceased but the left facial paralysis remained.

In a wound of the right *hip* a localized tetanus set in 7 days after the wound. There were twitchings first of the left and later, to a less extent, of the right lower limb. The twitchings continued until death, which occurred 2 days later. There was no trismus, opisthotonos, or spasms, except in the lower limbs.

A similar case, with a *knee* wound, was observed. Amputation of the left thigh was performed. The twitchings, without other symptoms, continued in both limbs until death, three days later.

In a case with a shell wound of the thigh and calf of the left leg, tetanus developed 14 days after injury. Tetanus serum had been administered 10 days before the wounds of the leg, because of a slight face wound, and the preventive dose was not repeated. There was at first only twitching of the left foot and leg. A week later there was no relaxation between the spasms. The tetanic rigidity continued for three months without other symptoms until the patient was removed and lost sight of.

In another case rigidity occurred in the right arm as a result of an entry wound at the vertebral end of the spine of the right scapula. The serum was administered the day following the wound and the symptoms first appeared upon the 22nd day. The tension might be relieved by forcibly extending the arm, but it immediately returned upon relaxation. The right trapezius and sternomastoid also twitched and contracted tonically. There was cutaneous anesthesia over the area of the 4th cervical nerve.

B. is led to suppose that local tetanus occurs only as a result of muscle injuries, and not subcutaneous ones.

B. thinks there is considerable advantage to be derived from early injections of serum into the muscles of the wounded limb. He thinks there is danger of anaphylaxis from secondary intravenous injections

